# The G-Protein β3 subunit 825 TT genotype is associated with epigastric pain syndrome-like dyspepsia

**DOI:** 10.1186/1471-2350-11-13

**Published:** 2010-01-26

**Authors:** Tadayuki Oshima, Shigemi Nakajima, Tetsuji Yokoyama, Fumihiko Toyoshima, Jun Sakurai, Junji Tanaka, Toshihiko Tomita, Yongmin Kim, Kazutoshi Hori, Takayuki Matsumoto, Hiroto Miwa

**Affiliations:** 1 Divisions of Upper Gastroenterology, Department of Internal Medicine, Hyogo College of Medicine, Nishinomiya, 663-8501, Japan; 2 Department of Medicine, Healthcare Social Insurance Shiga Hospital, Otsu, 520-0846, Japan; 3 Department of Human Resources Development, National Institute of Public Health, Wako, 351-0197, Japan; 4 Divisions of Lower Gastroenterology, Department of Internal Medicine, Hyogo College of Medicine, Nishinomiya, 663-8501, Japan

## Abstract

**Background:**

Although familial clustering of functional dyspepsia (FD) has been reported, the role of genetics in the susceptibility to FD is still not well understood. Several reports indicate an association between FD and G-protein β3 (GNB3) subunit gene polymorphism (C825T); however, these studies had small sample sizes and the findings are inconclusive. In the present study we clarified the association between GNB3 gene polymorphism and dyspepsia in a large population of Japanese subjects who visited a hospital for annual health check-up.

**Methods:**

Subjects with significant upper gastrointestinal findings were excluded. Subjects with dyspeptic symptoms were divided into either a postprandial distress syndrome (PDS) group or an epigastric pain syndrome (EPS) group according to the Rome III criteria. The presence of the GNB3 C825T polymorphism was then evaluated and logistic regression analysis was used to test all variables.

**Results:**

The GNB3 genotype distribution in subjects without dyspepsia was 191 CC (25.1%), 368 TC (48.4%), and 202 TT (26.5%) and 17 CC (25.0%), 29 TC (42.6%), and 22 TT (32.4%) in subjects with dyspepsia. No significant correlation was found between the GNB3 825TT genotype and dyspepsia. However, the TT genotype was significantly associated with subjects with EPS-like symptoms (odds ratio (OR) = 2.00, 95% confidence interval (CI); 1.07-3.76) compared to the CT/CC genotype adjusted for gender and age. No significant correlation was found between GNB3 polymorphism and PDS-like symptoms (OR = 0.68, 95% CI; 0.31-1.51). With the exclusion of subjects with both EPS- and PDS-like symptoms, only the TT genotype was significantly associated with EPS-like symptoms (OR = 2.73, 95% CI; 1.23-5.91).

**Conclusion:**

The homozygous GNB3 825T allele influences the susceptibility to EPS-like dyspepsia.

## Background

Functional dyspepsia (FD) is characterized by the presence of symptoms thought to originate in a gastroduodenal lesion in the absence of any organic or systemic disease that explains the symptoms [1]. The precise pathophysiology of the functional gastrointestinal disorders is still unknown, but several pathophysiological mechanisms have been described as possible etiological factors: visceral hypersensitivity [2,3], impaired proximal gastric accommodation [4], delayed gastric emptying [5], dysfunction of the autonomic nervous system [6] and underlying psychiatric disturbances [7]. Although risk factors for FD, including age, gender, *Helicobacter pylori *(*H. pylori*) infection, smoking and psychological disturbances, have also been reported, the data are not conclusive [8,9].

There is increasing evidence that susceptibility to functional gastrointestinal disorders is influenced by hereditary factors. G-protein β3 (GNB3) subunit gene polymorphism (C825T) is related to alternative splicing of the GNB3 protein and G-protein activity. As G-proteins play crucial roles as the ligands of G-protein coupling receptor (GPCR), this dysfunction interferes with intracellular signal transduction, and the 825T allele is associated with enhanced G-protein activation [10]. Studies indicate that the 825T allele of GNB3 (C825T) is associated with several diseases including essential hypertension [10]. Furthermore, individuals with TT and CT genotypes have a significantly higher sympathetic nervous system index and lower parasympathetic nervous system index than those with CC [11]. However, it is not likely that the C825T polymorphism in the GNB3 gene subunit is involved in mood disorder pathogenesis or depression [12,13]. In FD patients, 825CC, or CC and TT, genotypes are reported to be associated with dyspepsia [14,15]. On the other hand, the 825T allele is suggested to be related to dyspepsia in reports from Japan and the Netherlands [16,17].

Because of these conflicting findings among studies, we thought it necessary to undertake a large-scale general population study. In this study, we examined subjects who underwent an annual health check-up and investigated the relationship between the GNB3 gene (C835T) and dyspepsia in the Japanese population.

## Methods

### Subjects

People visiting Healthcare Center of Social Insurance Shiga Hospital for annual health check-up were asked to participate in the study. We estimate that 80% of those who underwent annual health check-up from December 2007 to April 2008 agreed to participate in the study. All subjects were Japanese. One thousand subjects completed an original self-administered questionnaire that assessed symptoms of dyspepsia, gastroesophageal reflux disease (GERD) and irritable bowel syndrome (IBS) according to the ROME III criteria. Dyspeptic symptoms were defined as pain or discomfort in the upper abdomen for the last 3 months, with symptom onset at least 6 months prior to the check-up. Postprandial distress syndrome (PDS)-like symptoms were defined as postprandial fullness and early satiation, and epigastric pain syndrome (EPS)-like symptoms were defined as epigastric pain and epigastric burning for more than 6 months with symptoms. Age, gender, previous upper gastrointestinal study, previous medication, smoking and alcohol consumption were also recorded. Subjects who consumed alcohol more than 3 days a week, regardless of the amount, were considered to have a drinking habit. Smoking was defined as smoking any number of cigarettes daily. One thousand blood samples were taken and 154 samples were excluded because of abnormal results in parameters including ALT or WBC. Upon subject request, imaging was performed by upper gastrointestinal barium study (UGI) in 350 subjects (325 controls, 25 FD cases) and esophagogastroduodenoscopy (EGD) in 136 subjects (118 controls, 18 FD cases), and 17 subjects were excluded due to the detection of definite and suspected organic diseases, including gastric and duodenal ulcers and gastric tumors. In total, 171 subjects (155 control and 16 FD) were excluded from the study based on abnormal findings. Among the 1000 subjects sampled, 318 controls and 25 dyspeptic subjects were uninvestigated by UGI or EGD. This study was approved by ethics committees of both the Hyogo College of Medicine and the Social Insurance Shiga Hospital and written informed consent was obtained from all participants.

### Genotyping

Genomic DNA was isolated from whole blood using the QIAamp DNA blood minikit (Qiagen, Hilden, Germany). Genotyping of the C825T polymorphism (rs5443) was performed in a blinded fashion with TaqMan SNP Genotyping Assays (C 2184734 10) using an ABI Prism 7900 Sequence Detection System (Perkin-Elmer Applied Biosystems, Foster City, CA, USA).

### Statistical Analysis

The distribution of age, body mass index (BMI), ALT or blood pressure (BP) was tested by the Kolmogorov-Smirnov test. The median and range in each group were calculated and differences were compared using the nonparametric Mann-Whitney U-test when necessary. Differences between GNB3 C825T genotypes and gender, smoking, or drinking were compared by Fisher's exact test. The distribution of alleles at each locus was assessed using the χ^2 ^statistic of the Hardy-Weinberg equilibrium. We compared GNB3 genotypes in subjects with abdominal symptoms vs. controls without symptoms, and assessed the association between specific types of symptoms and GNB3 C825T polymorphism. A logistic regression analysis was performed to test the influence of several factors in the association between the GNB3 C825T polymorphism genotype distribution and dyspepsia. A *P *< 0.05 was considered significant.

### Power of the Study

In this study, we assessed the potential association of symptoms of dyspepsia with GNB3 C825T allele status. In the healthy Japanese population, approximately 20% are expected to be homozygous for the 825T allele. Assuming that approximately 5% of subjects have symptoms of dyspepsia, a 20% increase in the prevalence of a genotype would be of clinical relevance. Thus, setting α = 0.05 and β = 0.80, 826 asymptomatic controls and 44 subjects with dyspepsia would be sufficient to identify a clinically relevant difference. The actual number of enrolled subjects (68 FD cases and 761 controls) has a power of 93% to detect the assumed difference. For the subgroup analyses for EPS (68 cases), PDS (43 cases), and EPS excluding PDS (28 cases), the power to assumed difference is 79%, 76%, and 61%, respectively.

## Results

Participant demographics and GNB3 C825T genotype distribution are summarized in Table 1. Age, BMI, ALT and BP (systolic and diastolic) did not follow normal distributions. The median age of the subjects with dyspepsia and the non-dyspeptic controls was 43 (range, 23-69) and 45 (range, 19-83) years. No significant bias was found between the groups for gender, age, smoking habit, drinking habit, BMI, ALT, systolic BP, or diastolic BP. The GNB3 genotype distribution in all subjects in this study was 208 CC (25.1%), 397 CT (47.9%), and 224 TT (27.0%), this distribution being compatible with the Hardy-Weinberg equilibrium (P = 0.228). The GNB3 genotype distribution in subjects without dyspepsia (controls) of 191 CC (25.1%), 368 CT (48.4%), and 202 TT (26.5%), and 17 CC (25.0%), 29 CT (42.6%), and 22 TT (32.4%) in subjects with dyspepsia was also compatible with the Hardy-Weinberg equilibrium. The distribution of the CC/CT/TT genotypes in the healthy controls was similar to other reports for Japanese populations [16,18]. The distribution of allele and genotype frequencies did not differ significantly between males and females.

**Table 1:** Participant demographics and genotype distributions

	Controls (n = 761)	FD, total (n = 68)	*P*^2^	EPS (n = 43)^4^	*P*^2^	PDS (n = 40)^4^	*P*^2^
Age (years)^1^	45 (19-83)	43 (23-69)	0.164	43 (23-69)	0.079	43 (23-64)	0.053
Sex (male%)	42.7	36.8	0.372	39.5	0.752	32.5	0.251
Smoking (%)	27.6	27.9	1.000	34.9	0.299	27.5	1.000
Drinking (%)	30.0	36.8	0.272	37.2	0.314	32.5	0.726
BMI (kg/m^2^)^1^	22.0 (15.6-37.1)	21.5 (17.4-29.3)	0.400	21.6 (17.4-29.3)	0.801	20.9 (17.4-29.3)	0.084
ALT (IU/L)^1^	18 (4-40)	17 (8-38)	0.478	18 (8-38)	0.965	17 (9-32)	0.189
Blood pressure (mmHg)^1^							
Systolic	118 (78-223)	117 (78-175)	0.735	116 (95-164)	0.994	115 (78-175)	0.282
diastolic	72 (42-126)	72 (48-104)	0.714	72 (49-104)	0.895	68 (48-104)	0.259
GNB3 genotype (%)							
CC (wild type)	25.1	25.0		18.6		25.0	
CT	48.4	42.6		39.5		42.6	
TT	26.5	32.4	0.546	41.9	0.113	32.4	0.546
*P *for HWE^3^	0.368	0.240		0.282		0.240	

^1^Data are shown as median (range).

^2^*P *vs. controls by Mann-Whitney U-test (for age, BMI, ALT, and blood pressure) or Fisher's exact test (for other variables).

^3^*P *for deviation from Hardy-Weinberg equilibrium (HWE).

^4^Fifteen subjects simultaneously had EPS- and PDS-like symptoms.

FD, functional dyspepsia; EPS, epigastric pain syndrome; PDS, postprandial distress syndrome; BMI, body mass index.

The association of genotype with the overall FD phenotype compared with asymptomatic controls was not statistically significant (*P *= 0.546). Forty-three subjects had predominantly EPS-like symptoms, whereas 40 had predominantly PDS-like symptoms; 15 subjects simultaneously had EPS- and PDS-like symptoms (Table 1). No association of genotype in subjects with the EPS or PDS phenotype compared to that in controls was detected (*P *> 0.05). The odds ratios of the GNB3 C825T CT and TT genotypes relative to the CC genotype for the phenotypes of FD, EPS, PDS, and EPS excluding PDS, are shown in Table 2. There were no significant associations detected for the CC genotype in each of the FD, EPS or PDS phenotypes. Of the genotypes, only the TT genotype was significantly associated with subjects with EPS-like symptoms (odds ratio (OR) = 2.00, 95% confidence interval (CI); 1.07-3.76) adjusted for gender and age (Table 2). However, there was no association of genotypes and subjects with PDS-like symptoms (OR = 0.68, 95% CI; 0.31-1.51). After excluding subjects with both EPS- and PDS-like symptoms, the TT genotype was significantly associated with EPS-like symptoms compared to the CT/CC genotype (OR = 2.73, 95% CI; 1.23-5.91) and compared to the CC genotype (OR = 3.32, 95% CI; 1.07-10.28) (Table 2). Comparison of the TT/CT genotype with the CC genotype showed no significant association for each phenotype.

**Table 2:** Risk of dyspepsia according to GNB3 genotypes

	FD, total (n = 68)			EPS (n = 43)^1^			PDS (n = 40)^1^			EPS excluding PDS (n = 28)		
								
	OR	(95%CI)	*P*	OR	(95%CI)	*P*	OR	(95%CI)	*P*	OR	(95%CI)	*P*
GNB3 genotype												
CC	1	reference		1	reference		1	reference		1	reference	
CT	0.85	(0.45-1.59)	0.605	1.03	(0.44-2.45)	0.943	0.70	(0.33-1.45)	0.335	1.31	(0.40-4.23)	0.657
TT	1.18	(0.61-2. 30)	0.618	2.05	(0.87-4.83)	0.102	0.54	(0.22-1.34)	0.185	3.32	(1.07-10.28)	0.037
*Dominant model*												
TT+CT vs. CC	0.97	(0.54-1.72)	0.910	1.39	(0.63-3.06)	0.414	0.64	(0.32-1.28)	0.207	2.03	(0.69-5.94)	0.196
*Recessive model*												
TT vs. CT+CC	1.32	(0.77-2.45)	0.310	2.00	(1.07-3.76)	0.030	0.68	(0.31-1.51)	0.342	2.73	(1.23-5.91)	0.008

^1^Fifteen subjects simultaneously had EPS- and PDS-like symptoms.

FD, functional dyspepsia; EPS, epigastric pain syndrome; PDS, postprandial distress syndrome;

OR, sex- and age-adjusted odds ratio, vs. 912 controls, by a multiple logistic regression model; CI, confidence interval.

## Discussion

This study evaluated GNB3 C825T polymorphism in a cohort comprising people with dyspepsia and healthy controls who visited a health care unit for annual health check-up. We demonstrated an association between homozygous GNB3 825T and EPS-like dyspepsia. Our data are consistent with other data from populations from Japan and the Netherlands. Tahara et al. reported that homozygous GNB3 825T status is associated with Japanese dyspeptic subjects without *H. pylori *infection [16], while van Lelyveld et al. reported that T allele carriers of GNB3 C825T polymorphism are associated with dyspepsia in a population in the Netherlands [17]. However, conflicting data have also been reported. Homozygous GNB3 825C status has been associated with unexplained dyspepsia in a German population [14]. Furthermore, meal-unrelated dyspepsia has been associated with both the homozygous GNB3 825T and C genotypes from the US [15]. These contrasting observations may be explained by differences in genotypic composition of populations in different countries, which comprise different racial groups. In fact, the frequency of 825TT is higher in Japanese people than in Caucasians. In addition, the definition of FD or sample selection may also affect the outcome. Moreover, the effect of type II error can not be excluded in relatively small sample sizes. In surveys of patients with uninvestigated dyspepsia, regular smoking has been identified as a risk factor in US populations [19]; however, smoking or drinking habits did not differ between the controls and FD subjects in the present study.

Involvement of the genotype for the generation of EPS-like symptoms demonstrated in this study may, in part, explain the previous findings of familial aggregation of patients with functional gastrointestinal disorders [20]. Studies have shown clustering of functional gastrointestinal disorders within families and studies of twins suggest that both genetic and environmental factors influence manifestation [20,21]. Hereditary factors of functional gastrointestinal disorders may be at least partially explained by the GNB3 polymorphism. If we can assume that people develop functional gastrointestinal disorders before they show symptoms, diagnostic testing may facilitate identifying disease clusters and treatment. Furthermore, studies suggest that the effects of medication can be directly or indirectly mediated through G-protein coupled receptors. Homozygous 825T allele status correlates with the greatest response to therapy with antidepressants [22].

It has been reported that several gene polymorphisms are involved in the development of IBS. There were only five subjects with IBS-like symptoms in the present study, and thus we could not analyze the involvement of GNB3 polymorphism in IBS and did not consider IBS in the analysis. De Vries et al. [23] reported that GERD is associated with the CT genotype of the GNB3 C825T polymorphism compared with the CC genotype. In the present study, 62 subjects with predominant symptoms of GERD were included. However, no association was found between the GNB3 C825T genotype and GERD symptoms. Furthermore, the analysis of data in the present study was not affected by concomitant GERD symptoms in some subjects.

Although the data in the present study are from one institute and from subjects undergoing annual health check-up, the frequency of the homozygous T genotype in the control group is consistent with other studies in Japan, confirming that the sample population is representative of the general Japanese population [16,18]. The majority of people who undergo health check-ups would have no symptoms but would have some concerns about their health. In the present, study 537 subjects (64.8%) did not have any symptoms and had normal blood examinations. Restricting the controls to those willing to undergo EGD or UGI tests may introduce major selection bias and psychosocial factors can influence the decision to have these tests. Therefore, we did not select subjects based on these examinations and included uninvestigated controls and dyspeptic subjects in the analysis. The relationship between subjects with ulcers or *H. pylori *infection and GNB3 polymorphism has not been reported, and the reported relationships between dyspepsia and diet, stress, anxiety, or *H. pylori *are not conclusive [24]. In the present study, subjects with ulcers were excluded from the study and all the analyzed subjects had normal blood screening findings.

Our data showed that the development of EPS-like symptoms is associated with the homozygous GNB3 825T genotype. Subjects with EPS-like symptoms are non-consulters, and thus it is reasonable to assume that these subjects represent specific subgroups of subjects with less severe functional disturbances. Consequently, the results may not be representative of all patients with functional gastrointestinal disorders. Further studies are needed to evaluate, in detail, the association between GNB3 polymorphism and symptoms in subjects who visit primary, secondary or tertiary care centers.

The higher prevalence of the 825TT genotype, which is related to enhanced signal transduction upon GPCR activity, may be involved in gastroduodenal motility and in the development of dyspeptic symptoms. Functional symptoms might result in part from genetically altered GPCR, which could in turn explain the multisystem manifestations commonly observed in these patients.

Tally et al. showed that up to 25% of the population experienced early satiety, nausea, upper abdominal pain, or discomfort, which are symptoms of FD, using a validated questionnaire [25]. We also investigated the association between GNB3 polymorphism and each of eight different types of dyspeptic symptoms. However, no correlation was found between GNB3 polymorphism and any of these symptoms (data not shown).

## Conclusions

We have demonstrated an association between EPS-like dyspeptic subjects and the GNB3 825TT genotype in the Japanese population. Further studies are needed to elucidate the specific signal transduction pathways affected by this genetic disorder.

## Abbreviations

FD: functional dyspepsia; *H. pylori*: *Helicobacter pylori*; GNB3: G-protein β3; PDS: postprandial distress syndrome; EPS: epigastric pain syndrome; OR: odds ratio; CI: confidence interval; GPCR: G-protein coupling receptor; GERD: gastroesophageal reflux disease; IBSL: irritable bowel syndrome; UGI: upper gastrointestinal barium study; EGD: esophagogastroduodenoscopy; BMI: body mass index; BP: blood pressure.

## Competing interests

The authors declare that they have no competing interests.

## Authors' contributions

TO participated in the design of the study, analyzed the data and wrote the paper. SN, together with JS, JT, TT, YK and KH, obtained the samples and the data. TY carried out the statistical analysis. FT analyzed the data and wrote part of the paper. TM supervised the research project and the drafting of the manuscript. HM was responsible for the conception of the study and designed the study. All authors approved of the final manuscript prior to submission.

## Acknowledgements

We thank Ms. Noriko Kamiya and Ms. Masayo Togawa for their excellent technical assistance.

## Pre-publication history

The pre-publication history for this paper can be accessed here:

http://www.biomedcentral.com/1471-2350/11/13/prepub
